# miR‐125a‐5p increases cellular DNA damage of aging males and perturbs stage‐specific embryo development via Rbm38‐p53 signaling

**DOI:** 10.1111/acel.13508

**Published:** 2021-11-09

**Authors:** Kuan Liang, Liangyu Yao, Shuxian Wang, Lu Zheng, Zhang Qian, Yifeng Ge, Li Chen, Xi Cheng, Rujun Ma, Chuwei Li, Jun Jing, Yang Yang, Wanwan Yu, Tongmin Xue, Qiwei Chen, Siyuan Cao, Jinzhao Ma, Bing Yao

**Affiliations:** ^1^ Center of Reproductive Medicine Nanjing Jinling Hospital The First School of Clinical Medicine Southern Medical University Nanjing China; ^2^ Department of Urology The First Affiliated Hospital of Nanjing Medical University Nanjing China; ^3^ Center of Reproductive Medicine Nanjing Jinling Hospital Clinical School of Medical College Nanjing University Nanjing China; ^4^ Basic Medical Laboratory Nanjing Jinling Hospital Clinical School of Medical College Nanjing University Nanjing China; ^5^ Department of Emergency medicine Jinling Hospital, Medical School of Nanjing University Nanjing China; ^6^ Department Reproductive Medical Center Jinling Hospital Nanjing Medicine University Nanjing China; ^7^ School of Life Science Nanjing Normal University Nanjing China

**Keywords:** DNA damage, male aging, miRNA, Rbm38, sperm

## Abstract

An increasing number of men are fathering children at an older age than in the past. While advanced maternal age has long been recognized as a risk factor for adverse reproductive outcomes, the influence of paternal age on reproduction is incompletely comprehended. Herein, we found that miR‐125a‐5p was upregulated in the sperm of aging males and was related to inferior sperm DNA integrity as an adverse predictor. Moreover, we demonstrated that miR‐125a‐5p suppressed mitochondrial function and increased cellular DNA damage in GC2 cells. We also found that miR‐125a‐5p perturbed embryo development at specific morula/blastocyst stages. Mechanistically, we confirmed that miR‐125a‐5p disturbed the mitochondrial function by targeting Rbm38 and activating the p53 damage response pathway, and induced a developmental delay in a p21‐dependent manner. Our study revealed an important role of miR‐125a‐5p in sperm function and early embryo development of aging males, and provided a fresh view to comprehend the aging process in sperm.

## Graphical abstract

miR‐125a‐5p was increased in the sperm of aging males. miR‐125a‐5p suppressed mitochondrial function and increased cellular DNA damage by targeting Rbm38 and activating p53 damage response pathway. miR‐125a‐5p perturbed embryo development at specific morula/blastocyst stages in a p21‐dependent manner.

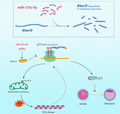

**Abbreviations**
APA: Advanced paternal age
DFI: DNA fragmentation index
OSA: Oxidative stress adducts
PR: Progressive motility
ROS: Reactive oxygen species
miRNAs: MicroRNAs
ATP: Adenosine triphosphate
PN: Pronuclei
CASA: Computer‐assisted sperm analysis
SCD: Sperm chromatin dispersion
MMP: Mitochondrial membrane potential
TESA: Testicular sperm aspiration
oxLDL: Oxidized low‐density lipoprotein
DMEM: Dulbecco's modified Eagle's medium
FBS: Fetal bovine serum
OTM: Olive tail moment

## 1 INTRODUCTION

Understanding how age affects fertility is becoming increasingly important, as couples are delaying childbearing toward later stages of their lives in many countries. It has long been known that women have a natural limit to their ability to conceive a child, with the window of their fertility limited by the availability of oocytes and the impending approach of menopause. However, men are seemingly untouched by the notion of this fertility precipice (Kovac et al., 2013). Although increasing maternal age is well established as a negative indicator of fertility, reproductive success, and offspring fitness, the influence of advanced paternal age (APA) on reproduction is poorly comprehended.

The relationship between paternal age and semen quality has been examined in multiple epidemiologic studies, and an age‐related decline in semen quality has been reported in numerous studies (Li et al., 2011). A systematic review using data from 90 studies (93,839 subjects) indicated that semen volume, percentage motility, progressive motility (PR), and normal morphology declined with age (Johnson et al., 2015). Recently, a retrospective cohort study of 16,945 semen samples indicated that the DNA fragmentation index (DFI) and oxidative stress adducts (OSA) increased with age, while the high DNA stainability declined with age (Vaughan et al., 2020). Inferior sperm parameters, especially increased sperm DFI, are often associated with undesirable embryo development and poor pregnancy outcomes.

The mechanisms responsible for age‐dependent patterns of decline in semen traits are not fully comprehended, but the damage from reactive oxygen species (ROS) is thought to be an important contributor (Johnson et al., 2015). ROS are produced in the mitochondria and their abnormal increase usually indicates mitochondrial damage or dysfunction. Spermatozoa have limited antioxidant defenses that are easily overwhelmed by excessive ROS, resulting in damage to the nucleus and mitochondrial DNA. Increased ROS levels are correlated with decreased sperm motility and accumulated DNA fragmentation in both the nucleus and mitochondria, which are in turn linked with heightened sperm dysfunction and abnormalities (Agarwal et al., 2008). Mitochondrial function regulation and ROS generation modification could be feasible strategies to adjust sperm fitness, including sperm motility and DFI.

Except for possible mutagenic events, aging is associated with widespread epigenetic changes, and epigenetic alterations in sperm are increasingly implicated in beneficial or deleterious effects on the sperm function, and embryo or offspring development (Milekic et al., 2015). MicroRNAs (miRNAs) are a class of small RNAs that perform essential functions as posttranscriptional regulators of gene expression in multiple tissues, and miRNAs in sperm could be typical mediators of epigenetic regulation and may participate in the modulation of sperm function. Numerous studies have proven that miRNAs, encoded by the nuclear or mitochondrial genome, could regulate the mitochondrial function (Zhang et al., 2019). Recently, miR‐125a‐5p was found to be able to induce mitochondrial dysfunction, increase ROS production, activate inflammasomes and pyroptosis, and promote the process of atherosclerosis (Zhaolin et al., 2019). Another study found that miR‐151a‐5p decreased mitochondrial respiratory activity and adenosine triphosphate (ATP) levels by targeting the mitochondrial transcription mt‐Cytb in asthenozoospermia (Zhou et al., 2015). These results suggest that miRNAs might modulate sperm function through a mitochondria‐dependent pathway.

Aging males are more susceptible to the accumulating harmful mutations and compounds. Numerous studies have identified APA as a risk factor for spontaneous abortion and may contribute to adverse reproductive outcomes, such as schizophrenia, autism, and several X‐linked recessive and autosomal dominant disorders (Brandt et al., 2019; Denomme et al., 2020; Johnson et al., 2015). The spermatozoa can deliver their RNA to oocytes at fertilization and function in specific instances, including protein translation, packaging of the paternal genome, and early embryo development. Many studies have found that sperm‐borne miRNAs are involved in embryo development (Liu et al., 2012). Microinjection of miRNAs into pronuclei (PN)‐stage embryos provides direct evidence that alterations of miRNA abundance in early embryo development could even induce phenotypes in adult offspring (Grandjean et al., 2009), and the microinjection of miRNAs dysregulated in the sperm by a father's chronic stress might cause targeted degradation of stored maternal mRNAs and induce a cascade of molecular events that ultimately perturb early embryo development (Rodgers et al., 2015). Therefore, we wondered whether the differentially expressed miRNAs in the sperm of aging males participate in the modulation of sperm function, or even contribute to early embryo development.

In order to investigate the expression patterns of advanced age on reproduction, our group previously performed high‐throughput sequencing of small RNAs in sperm, oocytes, and embryos of aged and young mice (Ma et al., 2020). Many miRNAs were found to be differentially expressed in sperm, oocytes, and embryos in aged and young mice. Excluding differentially expressed miRNAs in oocytes, and overlapping the specific miRNAs in sperm with the differentially expressed miRNAs in embryos, we obtained 33 miRNAs that might contain the contributor of embryo development from the sperm of aging males. Meanwhile, some of these miRNAs were associated with mitochondria, including miR‐574, miR‐128, let‐7b, miR‐24, and miR‐125a (Ma et al., 2020). In the previous study of our group, we found that miR‐574 was upregulated in the sperm of aging males and was related to poor sperm motility as an adverse predictor. MiR‐574 suppresses the mitochondrial function and reduces cellular ATP production by directly targeting mt‐ND5, and induces a downward trend in embryonic development. However, the role of miR‐125a‐5p, as a paternal factor, in the sperm of aging males or early embryo development is incompletely understood.

MiR‐125a‐5p is highly conserved between humans and mice, and has been reported to be associated with aging processes (Che et al., 2014; Dimitrakopoulou et al., 2015; Xu et al., 2017). Moreover, miR‐125a‐5p could induce mitochondrial dysfunction (Zhaolin et al., 2019), increase cellular ROS production (Chen et al., 2017), and might be involved in early embryo development (Byrne & Warner, 2008). In the present study, we sought to investigate the role of miR‐125a‐5p in sperm function and early embryo development, and address the mechanisms by which miR‐125a‐5p participates in the process of semen quality decrease or dysembryoplasia induced by male aging. We found that miR‐125a‐5p was upregulated in the sperm of aging males and was related to inferior sperm DNA integrity as an adverse predictor. Moreover, we confirmed that miR‐125a‐5p suppressed mitochondrial function and increased cellular DNA damage by targeting Rbm38 and activating the p53 damage response pathway in GC2 cells. Furthermore, we found that miR‐125a‐5p induced a developmental delay at specific morula/blastocyst stages in a p21‐dependent manner. Overall, we propose the important roles of miR‐125a‐5p in sperm function and early embryo development of aging males.

## 2 RESULTS

### 2.1 MiR‐125a‐5p was upregulated in the sperm of aging males and was related to poor sperm quality

**FIGURE 1:**
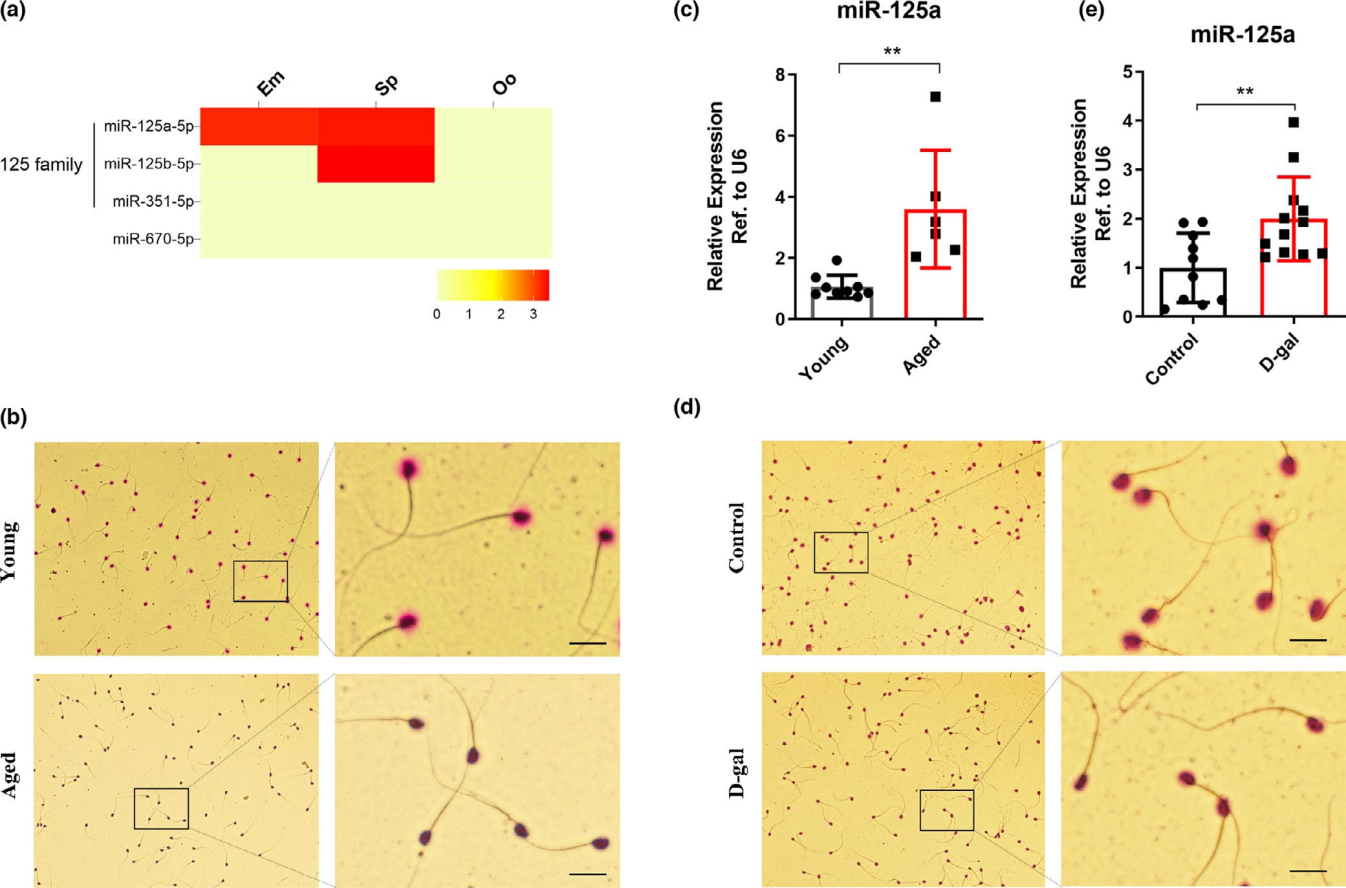
MiR‐125a‐5p was upregulated in the sperm of aging males and was related to poor sperm quality. (a) Expression of the miR‐125 family in the sperm (Sp), oocytes (Oo), and embryos (Em). (b) Representative photographs of the DFI in the sperm of the natural aging mouse model (detected by the SCD method), scale bar = 100 μm. (c) The expression of miR‐125a‐5p in the sperm of natural aging mouse model; a *t*‐test was used to assess the differences between the two groups. **p < 0.01; (d) Representative photographs of the DFI in the sperm of the D‐gal‐induced aging mouse model (detected by the SCD method), scale bar = 100 μm. (e) The expression of miR‐125a‐5p in the sperm of D‐gal‐induced aging mouse model; a *t* test was used to assess the differences between the two groups. **p < 0.01

In a previous study of our group, we found that numerous miRNAs, including miR‐574 and miR‐125a‐5p, were increased in the sperm of aging males. Subsequently, we demonstrated that mitochondria‐related miR‐574 could suppress mitochondrial function, reduce cellular ATP production by directly targeting mt‐ND5 in aging males, and have an adverse effect on early embryonic development (Ma et al., 2020). Moreover, we found that miR‐125a‐5p, which is highly conserved between humans and mice (Figure S1a), was also upregulated in both sperm and embryos of aging males (Figure 1a), suggesting that it may play a role in the sperm function or embryo development of aging males. Thus, we conducted a follow‐up study.

Two aging mouse models were established as described previously to explore the role of miR‐125a‐5p in the aging process of males. In the natural aging model, the body weight of aged mice was higher than that of young mice, but no significant difference in testicular weight was found between the two groups, leading to an obvious decrease in the testicular organ index (Figure S1b–d). The sperm parameters were assessed by computer‐assisted sperm analysis (CASA), and significant declines in sperm concentration, total motility, and PR were found in the aged group (Figure S1e–g). Subsequently, we analyzed sperm DFI by the Sperm Chromatin Dispersion (SCD) method and found a decline of halo sperm in the aged group, indicating that the sperm DFI was elevated in the aged group (Figure 1b, and Figure. S1h). We then detected the expression of miR‐125a‐5p in the sperm of the two groups and found that miR‐125a‐5p was significantly upregulated in the aged group (Figure 1c). Further analysis of the relationship between sperm parameters and miR‐125a‐5p expression revealed that miR‐125a‐5p expression was inversely related to sperm motility and progressive motility, especially to halo sperm cells in the aged group, but not to sperm concentration (Figure S1i–l).

We then established a D‐gal‐induced aging mouse model as described previously, by injecting D‐gal subcutaneously into the mice daily for 42 days. No significant differences in body weight, testicular weight, or testicular organ index were found between the D‐gal‐treated mice and the control mice (Figure S2a–c). Subsequently, we analyzed the sperm parameters of the two groups by CASA and found significant decreases in sperm concentration, total motility, and PR in the D‐gal‐treated group (Figure S2d–f), consistent with our expectations and similar to the observations in the natural aging models. We also observed a similar decline in halo sperm cells in the D‐gal‐treated group (Figure 1d, and Figure S2g). Thereafter, we detected the expression of miR‐125a‐5p in the sperm of the two groups and found that miR‐125a‐5p was significantly upregulated in the D‐gal‐treated group (Figure 1e). Further analysis of the relationship between sperm parameters and miR‐125a‐5p expression revealed that miR‐125a‐5p expression was inversely related to sperm motility, PR, and halo sperm cells, but not to sperm concentration (Figure S2h–k). Moreover, we collected clinical semen samples from the Reproductive Medicine Center of Nanjing Jinling Hospital and detected the expression of miR‐125a‐5p in the sperm of patients more than or less than 40 years old. We observed that miR‐125a showed an upward trend in the sperm of patients more than 40 years old (Figure S2l). It was considered that confounding factors other than age might be involved in the detection, and the fertility status of human patients might be variable and different from that of laboratory animals. Collectively, these experiments indicated that miR‐125a‐5p was upregulated in the sperm of aging males and was related to poor sperm quality, including DFI.

### 2.2 MiR‐125a‐5p impaired mitochondrial function and induced elevated DNA damage in GC2 cells

**FIGURE 2:**
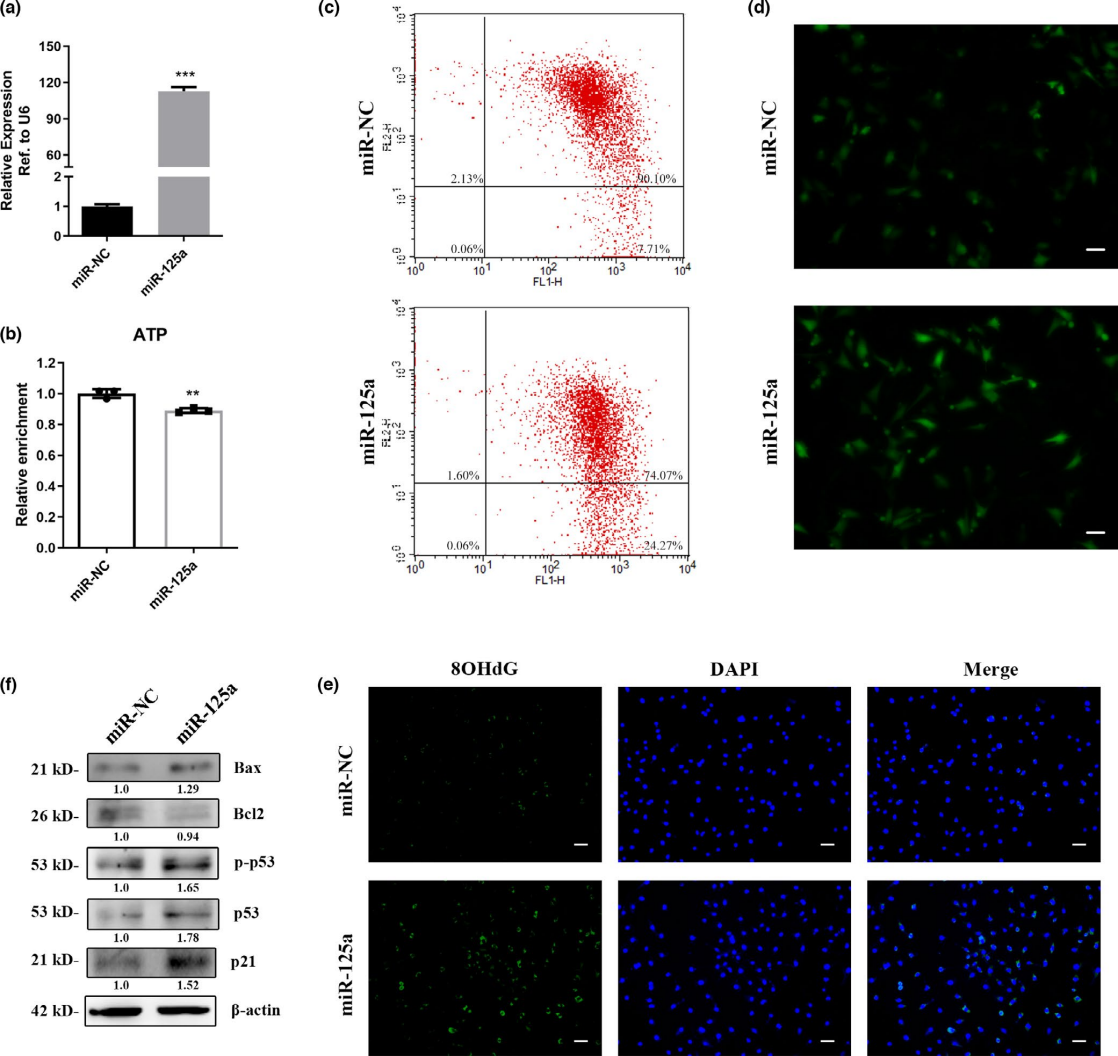
Overexpression of miR‐125a‐5p impaired mitochondrial function and induced DNA damage in GC2 cells. (a) miR‐125a‐5p expression in GC2 cells transfected with miR‐125a‐5p mimics and negative control. (b and c) ATP and MMP levels of GC2 cells transfected with miR‐125a‐5p mimics and negative control. (d) ROS levels of GC2 cells transfected with miR‐125a‐5p mimics and negative control. Scale bar = 100 μm. (e) 8‐OHdG (green) staining of GC2 cells transfected with miR‐125a‐5p mimics and negative control. The nuclei were stained blue with 4,6‐diamidino‐2‐phenylindole (DAPI). Scale bar = 100 μm. (f) The protein levels of Bax, Bcl2, p53 and p21 in the GC2 cells transfected with miR‐125a‐5p mimic and control

To identify the potential function of miR‐125a‐5p, miR‐125a‐5p mimics and negative control were transfected to GC2 cells for gain‐ or loss‐of‐function studies (Figure 2a). ATP production was obviously decreased after overexpressing miR‐125a‐5p (Figure 2b). Furthermore, flow cytometry was adapted to detect the mitochondrial membrane potential (MMP) of transfected GC2 cells. A significant increase in the ratio of Q4 district/Q2 district was observed in GC2 cells transfected with miR‐125a‐5p mimics (Figure 2c), suggesting that miR‐125a‐5p might lead to mitochondrial membrane potential abnormalities. Moreover, ROS and DNA damage levels (marked by 8‐OHdG and comet array) were detected in GC2 cells transfected with miR‐125a‐5p mimics. Our results demonstrated that miR‐125a‐5p significantly increased cellular ROS and DNA damage levels (Figure 2d–e, and Figure S3a,b).

We found that the sperm DFI was significantly increased in the naturally aged and D‐gal‐treated mice, consistent with the clinical observations that the DFI and OSA increased with age. Moreover, we found that miR‐125a‐5p impaired mitochondrial function and induced increased DNA damage in the GC2 cell model. The p53 cascade, which is the conventional signal involved in DNA damage responses, was measured in GC2 cells transfected with miR‐125a‐5p. As shown in Figure 2f, we found that the levels of p53 and p21 and the ratio of Bax/Bcl2, an indicator of mitochondrial dysfunction, were increased in the miR‐125a‐5p‐transfected group. Furthermore, we assessed the expression levels of OXPHOS, gamma H2AX, and damage repair genes 53BP1 and Aptx (Sykora et al., 2011) in GC2 cells transfected with miR‐125a‐5p, and found that OXPHOS levels were decreased, but gammaH2AX, 53BP1 and Aptx were significantly increased in the miR‐125a‐5p‐transfected group (Figure S3c,d). Together, these results suggested that miR‐125a‐5p impaired mitochondrial function and induced elevated DNA damage in GC2 cells and that the p53 pathway might be involved in this process.

### 2.3 MiR‐125a‐5p depletion relieved mitochondrial dysfunction and decreased cellular DNA damage

**FIGURE 3:**
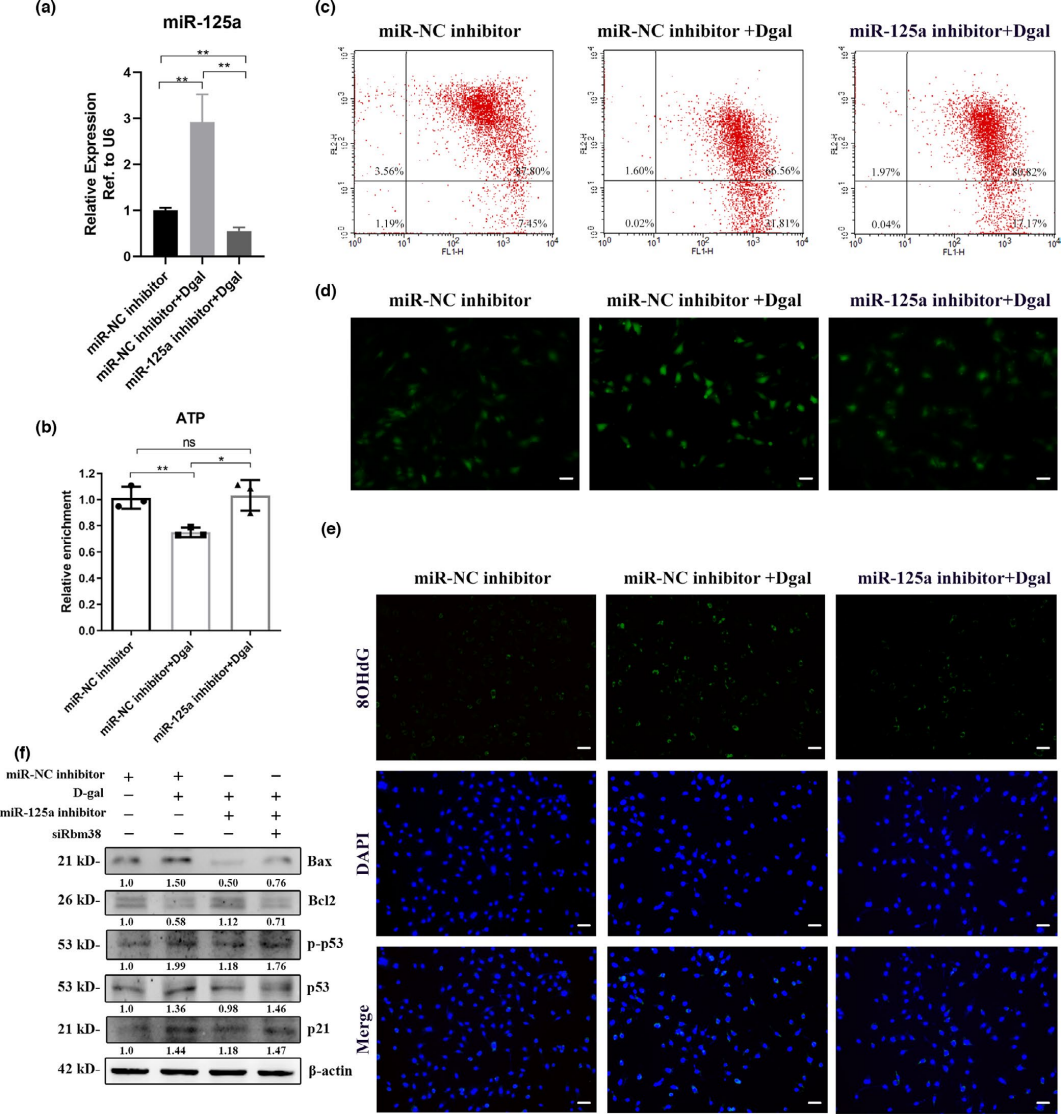
MiR‐125a‐5p depletion relieved mitochondrial dysfunction and decreased cellular DNA damage. (a) miR‐125a‐5p expression in GC2 cells transfected with miR‐125a‐5p inhibitor or negative control with D‐gal (50 mmol/L) supplementation. (b and c) ATP and MMP levels of GC2 cells transfected with miR‐125a‐5p inhibitor or negative control with D‐gal supplementation. (d) ROS levels of GC2 cells transfected with miR‐125a‐5p inhibitor or negative control with D‐gal supplementation. Scale bar = 100 μm. (e) 8‐OHdG staining (green) of GC2 cells transfected with miR‐125a‐5p inhibitor or negative control with D‐gal supplementation. The nuclei were stained with DAPI. Scale bar = 100 μm. (f) The protein levels of Bax, Bcl2, p53, and p21 in the GC2 cells treated as shown

We treated GC2 cells with D‐gal and found that D‐gal increased the expression of miR‐125a‐5p (Figure 3a). To further explore the role of miR‐125a‐5p, we reduced the expression of miR‐125a‐5p in GC2 cells by transfection with a miR‐125a‐5p inhibitor (Figure 3a). Then, we measured the cellular ATP levels and found that D‐gal decreased cellular ATP production in GC2 cells and that the miR‐125a‐5p inhibitor alleviated this decrease (Figure 3b). Moreover, the results of MMP showed that D‐gal increased the ratio of Q4 district/Q2 district and that the miR‐125a‐5p inhibitor mitigated this effect (Figure 3c). Furthermore, ROS and DNA damage levels were detected in GC2 cells treated with D‐gal or miR‐125a‐5p inhibitor. We observed that D‐gal significantly increased cellular ROS and DNA damage levels, and the miR‐125a‐5p inhibitor could relieve these increases (Figure 3d‐e, and Figure S3e,f). Similarly, we examined the level of p53 signals in these groups, and found that the levels of p53 and p21 and the ratio of Bax/Bcl2 were significantly decreased in the miR‐125a‐5p inhibition group compared with the D‐gal treatment group (Figure 3f). We assessed the expression levels of OXPHOS, gamma H2AX, and damage repair genes 53BP1 and Aptx, and found that D‐gal decreased OXPHOS levels and increased gamma H2AX, 53BP1, and Aptx levels in GC2 cells, and that the miR‐125a‐5p inhibitor alleviated these effects. (Figure S3d,g). Collectively, the results indicated that miR‐125a‐5p depletion could relieve mitochondrial dysfunction and decrease cellular DNA damage, and the p53 pathway might play a role in this process.

### 2.4 MiR‐125a‐5p regulated cellular DNA damage via Rbm38‐p53 signaling

**FIGURE 4:**
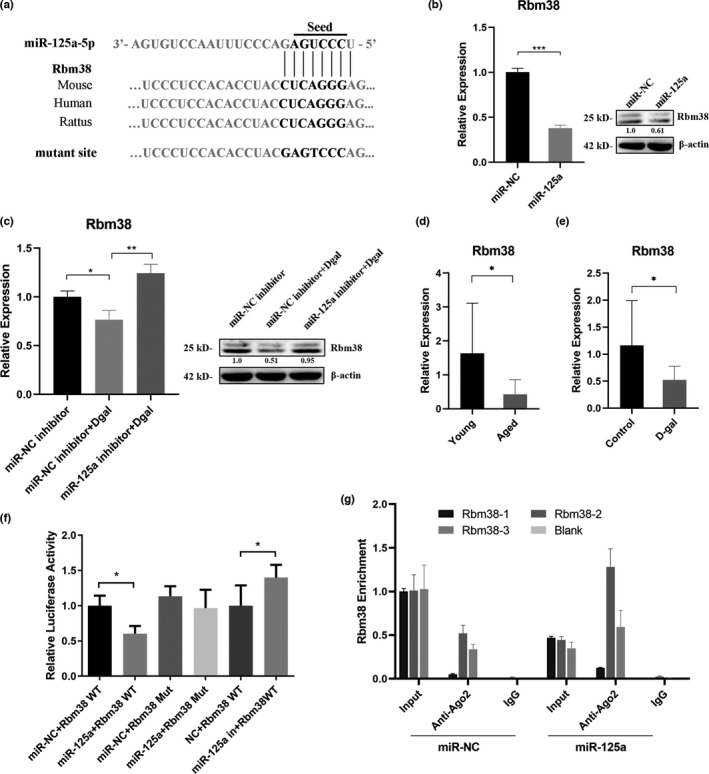
MiR‐125a‐5p regulated cellular DNA damage *via* the Rbm38‐p53 signaling pathway. (a) The putative site at which miR‐125a‐5p binds to Rbm38 in multiple species. The mutant vector was constructed by mutating miR‐125a‐5p‐binding sites in Rbm38. (b and c) mRNA and protein levels of Rbm38 in GC2 cells transfected with miR‐125a‐5p mimics/inhibitor or their respective controls. ***p < 0.001, **p < 0.01, *p < 0.05. (d–e) The expression of Rbm38 in the sperm of the natural aging mouse model and D‐gal‐induced aging mouse model. (f) Luciferase activity in GC2 cells cotransfected with miR‐125a‐5p mimics/inhibitor or their respective controls and the WT/Mut luciferase reporter vector. (g) Anti‐Ago2 RIP was performed in GC2 cells transfected with miR‐125a‐5p or negative control, followed by qRT‐PCR to detect Rbm38 associated with Ago2

Since miR‐125a‐5p was involved in the regulation of mitochondrial function, we predicted 314 candidate targets of miR‐125a‐5p within the mitochondrial pathway (Schaum et al., 2020) using the bioinformatics tools TargetScan, miRanda, and miRWalk (Table S2), and selected Mark2, Loxl2, and Rbm38 to investigate their expression pattern in miR‐125a‐5p‐treated cells, after surveying the literature of these genes. We found that the RNA‐binding motif protein 38 (Rbm38) gene, containing an evolutionarily conserved target site of miR‐125a‐5p (Figure 4a), exhibited a fine tendency upon miR‐125a mimics or inhibitor treatment (Figure S3i). Rbm38 was found to decrease during aging within the associated genes, which were clustered in consistent trajectory groups with coherent biological functions, including mitochondrial function, in the bulk RNA sequencing of multiple tissues (Schaum et al., 2020). Rbm38 was necessary for suppressing accelerated aging, and mice deficient in Rbm38 exhibited signs of accelerated aging and enhanced accumulation of p53 via the Rbm38‐p53 loop by modulating p53 translation (Zhang et al., 2014). Based on this, we then selected Rbm38 as a candidate target of miR‐125a‐5p and detected its mRNA and protein expression in GC2 cells transfected with miR‐125a‐5p mimics or inhibitor. We found that the mRNA and protein levels of Rbm38 were significantly lower in cells transfected with miR‐125a‐5p mimics and higher in cells transfected with miR‐125a‐5p inhibitor than in cells transfected with negative control (Figure 4b,c). Furthermore, we tested whether the upregulation of miR‐125a‐5p would reduce the expression of Rbm38 in the sperm of the two aging models. As we assumed, the expression of Rbm38 was markedly downregulated in the sperm of the two aging groups (Figure 4d,e).

To verify the relationship between miR‐125a‐5p and Rbm38, we constructed luciferase reporters containing either the wild‐type (WT) or mutated (Mut) miR‐125a‐5p‐binding sites (seed sequence) of Rbm38 (Figure 4a). Overexpression of miR‐125a‐5p reduced the luciferase activity of the WT reporter vector but not that of the Mut reporter vector, suggesting that miR‐125a‐5p directly binds to Rbm38 (Figure 4f). It is known that miRNAs bind to their targets and cause translational repression and/or RNA degradation in an Ago2‐dependent manner. To determine whether Rbm38 is regulated by miR‐125a‐5p in this manner, we conducted anti‐Ago2 RIP in GC2 cells transiently overexpressing miR‐125a‐5p. The Rbm38 pulldown by Ago2 was more highly enriched in miR‐125a‐transfected cells, suggesting that miR‐125a‐5p could target Rbm38 (Figure 4g). Moreover, we observed that knockdown of Rbm38 decreased OXPHOS levels, increased the levels of p53 and p21 and the ratio of Bax/Bcl2 (Figure 3f), and elevated the cellular DNA damage, gamma H2AX, 53BP1, and Aptx in GC2 cells (Figure S3d–g,h). Together, these results indicated that miR‐125a‐5p regulated cellular DNA damage *via* the Rbm38‐p53 response pathway.

### 2.5 miR‐125a‐5p induced a developmental delay at the morula/blastocyst stages in a p21‐dependent manner

**FIGURE 5:**
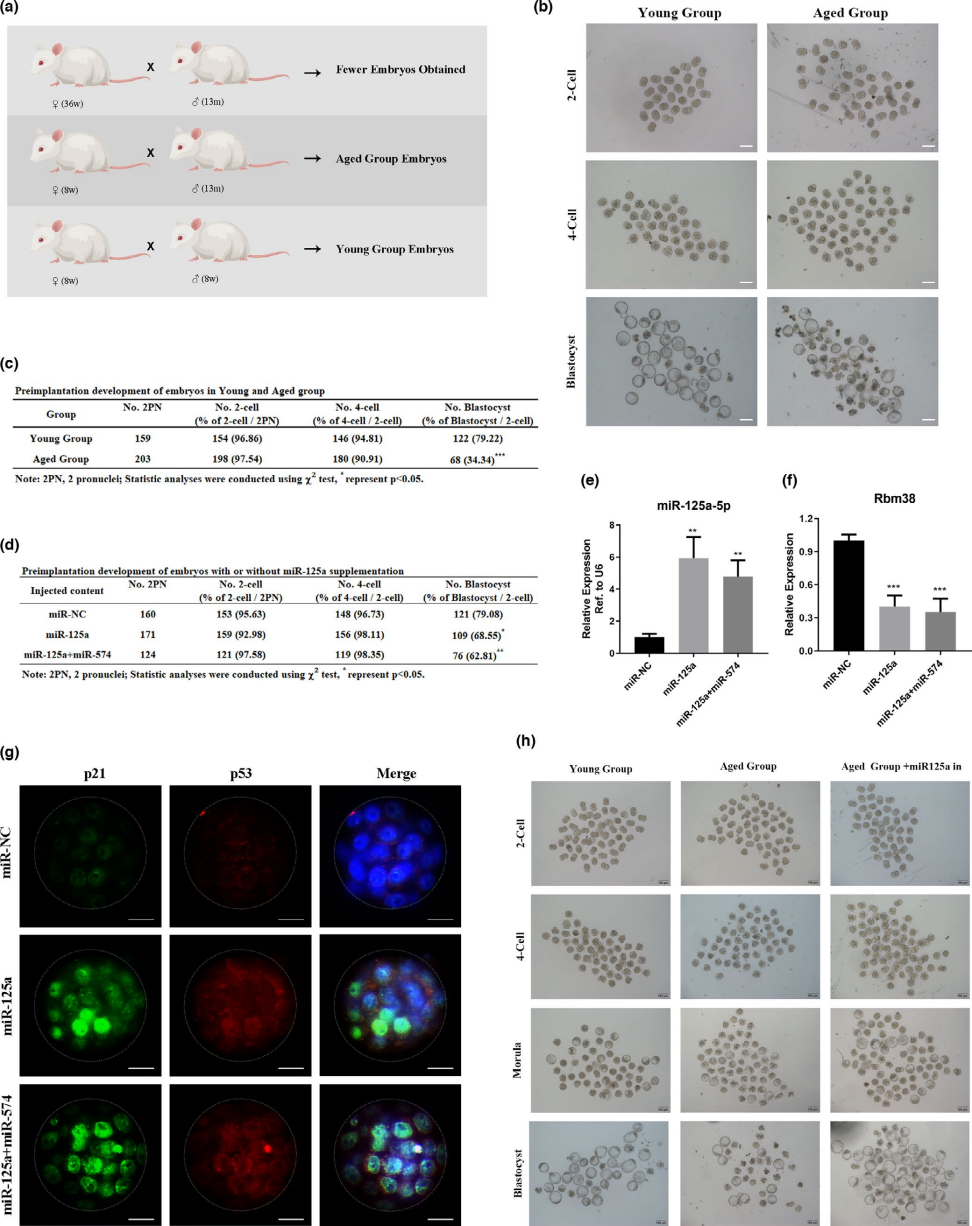
miR‐125a‐5p induced a developmental delay at the morula/blastocyst stages in a p21‐dependent manner. (a) The mating strategies of mice used in this study. (b and c). Representative photographs and the rate of preimplantation embryo development in the Aged and Young groups. Scale bar = 100 μm. (d) The rate of preimplantation embryo development with or without miR‐125a/miR‐574 supplementation. (e and f) The expression of miR‐125a‐5p and Rbm38 in the morulae with or without miR‐125a/miR‐574 supplementation. (g) Representative microscopic views of p21 (green) and p53 (red) in the morulae with or without miR‐125a/miR‐574 supplementation. Scale bar = 20 μm. (h) Representative views of preimplantation development of Aged and Young group embryos with or without miR‐125a‐5p inhibitor supplementation. Scale bar = 100 μm

Previous studies have proved that miRNAs might play a role in early embryonic development, and aging males exert certain levels of fertility reduction and early embryonic dysplasia (Katz‐Jaffe et al., 2013). Therefore, we wondered whether sperm miRNAs from aging males could play a role in early embryonic development. In order to test the influence of male aging on early embryonic development, special mating strategies were adopted, including Aged♂× Aged♀, Aged♂× Young♀, and Young♂× Young♀ (Figure 5a). We collected the zygotes of these groups, cultured them *in vitro* and found that the Aged♂× Aged♀ group obtained fewer zygotes to form embryos. The Aged♂× Young♀ group (Aged group embryos) and the Young♂× Young♀ group (Young group embryos) were thus chosen to survey the effect of male aging on early embryonic development. We found that the blastocyst formation, but not the two‐ or four‐cell formation, of the Aged group embryos was significantly decreased compared with that of the Young group embryos, suggesting that APA exhibited a negative effect on early embryonic development (Figure 5b,c). Then, we tested the expression of miR‐125a‐5p and Rbm38 in the blastocysts of the Aged group embryos and Young group embryos, and found that there was an increase in miR‐125a‐5p, but a decrease in Rbm38, in the Aged group compared with the Young group (Figure S4a,b).

In order to investigate the role of miR‐125a‐5p in APA‐induced embryo development delay, we then collected the Young group zygotes and overexpressed miR‐125a‐5p by microinjecting a miR‐125a‐5p mimic into zygotes. A similar experiment was performed with miR‐574, another miRNA that has been shown to suppress the mitochondrial function and influence early embryonic development, in our previous study (Ma et al., 2020). We added an experimental group that was microinjected with mixed miR‐125a/miR‐574, considering the multi‐microRNA‐harboring complex synergy in the regulation of early embryonic development. Our results indicated that the group microinjected with miR‐125a‐5p showed a significant downward trend in blastocyst formation, and the group injected with mixed miR‐125a/miR‐574 exhibited a more marked decreasing tendency, with no influence on the two‐ or four‐cell formation (Figure 5d). Further analysis of the morula formation in these groups showed that the microinjection of miR‐125a‐5p or mixed miR‐125a/miR‐574 did not affect the morula formation, suggesting that miR‐125a‐5p induced a developmental delay at the morula/blastocyst stages (Figure S4c). We then detected the expression of miR‐125a‐5p and Rbm38 in morulae and blastocysts. Similar to the results of the aged groups, miR‐125a‐5p was significantly increased in the microinjection group, and Rbm38 was markedly decreased in the microinjection group (Figure 5e,f; and Figure S4d,e).

Recent studies have reported that p21, a conventional target of the p53 pathway, is the key regulatory molecule involved in cell cycle arrest at the morula/blastocyst stages upon DNA damage from irradiated sperm to early embryos (Adiga et al., 2007). Ock et al.  found that zygotes exposure to 1‐ or 3‐Gy did not affect development to the morula stage; however, a significant number of morulae failed to develop to the blastocyst stage. p21 plays a vital role in cellular senescence during early embryonic development, and exposure to cellular stressors results in the upregulation of p21 in embryos (Ock et al., 2020). As we had confirmed that p21 and p53 signaling were activated in miR‐125a‐5p‐transfected GC2 cells, we wondered whether p21 was activated in the morulae and participated in morula/blastocyst arrest. Then, we used immunofluorescence microscopy to examine the expression and subcellular localization of p21 in morulae microinjected with miR‐125a‐5p, and found increases in the expression of p21 protein and nuclear localization in the miR‐125a‐5p microinjection group compared to the control group (Figure 5g). Moreover, we found that morula formation was constantly decreased in the Aged group compared with the Young group (Figure S4f), while the p21 expression was increased in the morulae of the Aged group compared with the Young group (Figure S4g). By combining the analyses of the expression of miR‐125a‐5p and Rbm38, we considered that the early embryonic development was a multistage and multimolecule participation process, and that miR‐125a‐5p might be involved in the specific regulation of morula/blastocyst stages in a p21‐dependent manner. Furthermore, we microinjected a miR‐125a‐5p inhibitor into the Aged group zygotes, and found that the morula and blastocyst formation were mildly increased (Figure 5h, and Figure S4h). Additionally, we assessed the expression of miR‐125a‐5p in the fetuses developed from the Aged or Young group embryos, and observed that miR‐125a‐5p showed no significant differences in multiple tissues of the fetuses developed from the Aged and Young group embryos, suggesting that miR‐125a‐5p might play a limited role in the development from embryo to fetus (Figure S4i). Overall, these results indicated that miR‐125a‐5p could participate in the specific stage regulation of early embryonic development, and induce a developmental delay at morula/blastocyst stages in a p21‐dependent manner.

## 3 DISCUSSION

Delayed fatherhood is becoming socially acceptable in many countries with the aging male population rapidly increasing, and issues of infertility are encountered more frequently. The overall decline in reproductive potential with advanced maternal age has been well documented in the scientific literatures. In contrast, limited information is available regarding the impact of paternal age on reproductive success. Increasing evidence suggests that APA is associated with declines in fertility and offspring fitness, independent of maternal age. Multiple effects of aging on sperm concentration, sperm motility, and DFI indicate that the quality of spermatozoa declines over time, but few studies have shed light on the molecular mechanisms that hamper sperm function in older men.

Sperm DNA integrity is important for men preparing for childbearing, because the damaged DNA may be transmitted to the offspring when the DFI levels exceed the DNA repair capacity of the oocyte (Rosiak‐Gill et al., 2019). Mitochondria is very important for sperm quality, and the homeostasis imbalance of the different hallmarks of aging (López‐Otín et al., 2013), such as “mitochondrial dysfunction”, “comprised autophagy”, and “DNA damage/genomic instability”, may all be involved in affecting the quality of sperm during aging (Fang et al., 2017; Scheibye‐Knudsen et al., 2015). Age‐related factors may be attributed to older men producing more sperm with DNA fragmentation. The reason for this could be a higher exposure to DNA‐damaging oxidative stress in their reproductive tracts or the increasing intracellular ROS generation during spermatogenesis resulting in apoptotic dysfunction in older men (Evenson et al., 2020). Extensive studies indicate that abnormal paternal factors, particularly sperm DNA and chromatin, have been demonstrated to be correlated with reduced embryo development, increased risk of miscarriage, and decreased successful conception (Robinson et al., 2012). For the assisted reproductive technologies, natural and IUI fertilization is likely to be successful in patients with <25% DFI, whereas patients with >25%–30% DFI should be moved to ICSI. At the >40% DFI level, which is commonly seen in men more than 50 years old, even the testicular sperm aspiration (TESA) is required (Evenson et al., 2020). A significant age‐dependent increase in sperm DFI might have potential clinical importance.

In this study, we found that miR‐125a‐5p was upregulated in the sperm of two aging mouse models and was related to elevated sperm DFI (Figure 1, and Figures S1–S2). Moreover, we proved that miR‐125a‐5p suppressed mitochondrial function, reduced cellular ATP production, and increased cellular ROS and DNA damage levels in GC2 cells (2, 3, and Figure S3). Mechanistically, we demonstrated that miR‐125a‐5p regulated mitochondrial function by targeting Rbm38 and activating the p53 damage response pathway (Figure 4). Furthermore, we evaluated the effects of miR‐125a‐5p on early embryo development and found that miR‐125a‐5p microinjection induced an embryo developmental delay at the morula/blastocyst stages in a p21‐dependent manner (Figure 5, and Figure S4).

MiR‐125a‐5p has been reported to be involved in the processes of aging in previous studies. Xu and colleagues found that miR‐125a‐5p was increased in the aging thymus and participated in age‐related thymic involution by targeting FoxN1 (Xu et al., 2017). MiR‐125a‐5p and numerous miRNAs were associated with cardiac aging and exhibited a synergistic effect (Dimitrakopoulou et al., 2015). In addition, miR‐125a‐5p was upregulated in old endothelial cells and impaired endothelial cell angiogenesis via targeting RTEF‐1 (Che et al., 2014). Moreover, miR‐125a‐5p was reported to be involved in mitochondrial dysfunction and ROS generation. Zeng et al. found that miR‐125a‐5p was increased in hyperlipidemic‐hyperglycemic conditions, implicated in oxidized low‐density lipoprotein (oxLDL)‐induced vascular endothelial cells pyroptosis, and caused mitochondrial dysfunction and ROS increases by targeting TET2 (Zhaolin et al., 2019). MiR‐125a‐5p could also be upregulated by curcumin, and increase ROS and decrease cell viability in osteosarcoma by inhibiting ERRa (Chen et al., 2017). In the reproductive system, Zhao and colleagues found that miR‐125a‐5p was increased in preeclampsia placental tissues compared with normal subjects, and miR‐125a‐5p mimics decreased HTR8/SVneo cell migration, proliferation, and angiogenesis abilities, and induced more cell arrest in the S stage (Xueya et al., 2020). Kim et al. observed that the miR‐125‐5p family was an important regulator of the expression and maintenance of maternal effect genes during early embryo development. They found that microinjection of miR‐125 family members would suppress the expression of Sebox and Lin28a and impair early embryogenesis, resulting in the arrest of embryogenesis at the two‐cell stage (Kim et al., 2016). However, in the present study, we found that microinjection of miR‐125a‐5p would trigger the early embryo development arrest at the morula/blastocyst stage (Figure 5). The difference in these results may come from the differences in microinjection dosage and embryonic generation strategy. In Kim's study, the PN embryos used for microinjection were derived from GV oocytes, and the microinjection dosage was 10 pl of 2 μM miRNA mimics. However, the zygotes used in our study were obtained with an in vivo method, and the microinjection used was at a much lower dosage (i.e., 2.5 pl of 100 nM) miRNA mimics. We thought that a low‐dosage microinjection might reflect the impact of miRNA from sperm on embryonic development more realistically.

Nixon et al.   found that sperm miRNA signature is influenced by their prolonged maturation within the male reproductive tract, and miR‐125a‐5p is highly expressed in the caput but reduces in the corpus and cauda epididymis (Nixon et al., 2015). Another group found that small RNAs are trafficked from epididymis to developing mammalian sperm (Sharma et al., 2018), and small RNAs gained during epididymal transit of sperm are essential for embryonic development in mice (Conine et al., 2018). However, miR‐125a‐5p showed no significant difference between cauda and caput sperm, and its function in embryo development has been not evaluated. In order to detect the expression pattern of miR‐125a‐5p in cauda and caput sperm of the aged and young mice, we isolated the sperm in cauda and caput and tested the expression of miR‐125a‐5p in them. We found that miR‐125a‐5p expression showed no significant difference between the cauda and caput sperm of the young mice, but increased markedly in the cauda and caput sperm of the aged mice compared with the young (Figure S5a), indicating that epididymal transit might be one reason for the miR‐125a‐5p increase in the sperm of aged mice. As we found that miR‐125a‐5p was increased in the caput of the aged mice compared with the young (Figure S5a), we also isolated mice testicular germ cells by a Hoechst‐FACS method (Figure S5b), measured the expression of miR‐125a‐5p in the haploid (1C) and tetraploid (4C) cells, and found that miR‐125a‐5p was upregulated in the haploid and tetraploid cells of aged mice (Figure S5c). These results indicated that the spermatogenesis process and the post‐testicular maturation might contribute to the increased expression of miR‐125a‐5p in the sperm of aging males.

Rbm38 was first discovered as a p53 family target, and later studies revealed the pleiotropic roles of Rbm38 in diverse pathological conditions *via* frequently forming a negative feedback loop with the p53 family (Zhang et al., 2011). Rbm38 is considered an intergenic suppressor in aging (Jiang et al., 2018) and decreases during aging in bulk RNA sequencing of multiple tissues (Schaum et al., 2020). Moreover, Rbm38 deficiency exhibited signs of accelerated aging and enhanced accumulation of p53 via the Rbm38‐p53 loop by modulating p53 translation (Zhang et al., 2014). p53 plays a crucial role in the regulatory response to cellular stress‐induced DNA damage. The activation of p53 by DNA damaging stressors is followed by a spectrum of responses such as apoptosis, cell cycle arrest, DNA repair, and the modulation of intracellular ROS (Torchinsky & Toder, 2010). Hu et al. found that H_2_O_2_ could activate p53 and regulate p53 target genes to affect early embryo development by modulating the expression of GADD45a and p21 (Hu et al., 2017). Recent studies have reported that p21 plays a vital role in cellular senescence during early embryonic development, and induces cell cycle arrest at the specific morula/blastocyst stages upon DNA damage from irradiated sperm to early embryos (Adiga et al., 2007; Ock et al., 2020). In the present study, we considered that miR‐125a‐5p might participate in the specific stage regulation of early embryonic development, and induce a developmental delay at morula/blastocyst stages in a p21‐dependent manner.

In our study, we confirmed the increase of miR‐125a‐5p in the sperm of two aging mouse models and observed a negative relationship between sperm DFI and miR‐125a‐5p. However, no such trend was observed in the clinical semen samples, suggesting that confounding factors other than age affected the detection. The cut‐off for APA used in this study was over 40 years old at the time of conception (Toriello & Meck, 2008), and further studies should be conducted with more stratification by age and on extensive semen samples, in addition to the samples from men attending infertility clinics. Herein, we proved the important role of miR‐125a‐5p on the sperm function of aging males and embryonic development; knockout or knockin mice should be established to test this hypothesis in further investigations. Moreover, we observed that the embryos microinjected with miR‐125a‐5p mimics showed a decreasing trend of embryonic development. Further investigation and longitudinal studies are required to examine the effects of pools of multiple miRNAs on early embryonic development. Furthermore, we evaluated the expression of miR‐125a‐5p in the fetuses developed from the Aged or Young group embryos, and found that miR‐125a‐5p exhibited no significant differences in multiple tissues of the fetuses developed from the Aged and Young group embryos, despite the increased expression of miR‐125a‐5p in the Aged group blastocysts, suggesting that the function of miR‐125a‐5p in the development from embryo to fetus needs to be further studied in future investigations.

In summary, our study delineates a miR‐125a‐Rbm38‐p53 module that regulates mitochondrial function and the DNA damage response with respect to functional implications in the sperm and early embryo development of aging males (Figure S5d). This suggests that miR‐125a‐5p might play an important role in sperm function and early embryo development, and the regulatory signaling might offer a fresh view to comprehend the aging process in sperm.

## 4 EXPERIMENTAL PROCEDURES

### 4.1 Animals and cell lines

Six to eight‐week‐old male C57BL/6 mice were purchased from Beijing Vital River Laboratory Animal Technology Co., Ltd., and housed in a 12‐h light:12‐h dark cycle at 22 ± 2°C with free access to food and water. The animal experiments were approved by the Ethics Committee of the Nanjing Jinling Hospital. Two aging mouse models were established as described in our previous study (Ma et al., 2020). D‐gal (Sigma‐Aldrich) (120 mg/kg/day) was injected subcutaneously into the mice daily for 42 days to construct the D‐gal‐induced aging mouse model, and saline was administered subcutaneously at the same volume in the control group (Wang et al., 2018). In the natural aging model, aged mice were raised routinely for more than 12 months as the aged group, and 6–8‐week‐old mice were raised as the young group (Miranda et al., 2018). Each group contained more than five mice at the end of the detection.

GC2 (ATCC catalog number CRL‐2196) was purchased from ATCC (Rockville) and cultured in high‐glucose Dulbecco's modified Eagle's medium (DMEM) supplemented with 10% fetal bovine serum (FBS) under 5% CO_2_ at 37°C.

### 4.2 Quantitative real‐time PCR

Total RNA was isolated from sperm or GC2 cells using a Total RNA Isolation Kit (BEI‐BEI Biotech), and from embryos using an RNA Isolation Kit (cat. no., KIT0204; Invitrogen; Thermo Fisher Scientific, Inc.). For miRNA detection, complementary DNA (cDNA) was synthetized from total RNA using miRNA‐specific primers (RiboBio). For mRNA detection, cDNA was synthetized from total RNA using PrimeScript^®^ RT Master Mix (Takara) reverse transcriptase, according to the manufacturer's instructions. cDNA was quantitated using RT‐qPCR with a Roche LightCycler^®^ 96 Real‐time PCR system (Roche Diagnostics). Real‐time PCRs were performed in triplicate. β‐actin and U6 were used as endogenous controls for mRNA and miRNA, respectively. Relative expression was calculated using the comparative ∆∆Ct method. The primers for miRNA real‐time PCR were purchased from RiboBio Company. The other primer sequences are presented in Table S1.

### 4.3 Alkaline comet assay

Alkaline comet assay was performed as previously described (Sykora et al., 2013). Cells were pretreated with miR‐125a‐5p mimics or inhibitor, with or without siRbm38 supplementation, following which they were incubated for the indicated time periods, trypsinized, washed, and resuspended in ice‐cold PBS. Cell suspension was embedded in 120 μl low‐melting point agarose (0.5% in dH_2_O at 37^o^C) onto agarose‐coated (1.5% in PBS) and dried slides that were submersed for 1 h in precooled lysis buffer (2.5 M NaCl, 100 mM EDTA, 10 mM Tris HCl, and 1% N‐laurylsarcosine, pH = 10 for alkaline comet assay); before cooling, 1% Triton was added to the lysis buffer. Slides were denatured and equilibrated for 20 min in precooled running buffer (300 mM NaOH, 1 mM EDTA, pH > 13 for alkaline comet assay). Following the denaturation step, slides were electrophoresed at 0.8 V/cm (300 mA for the alkaline version) for 22 min at 4°C. In the case of the alkaline version, the slides were neutralized three times and further (both for the neutral and alkaline comet assays) slides were rinsed in water, fixed in 100% ethanol, dried, and stained with propidium iodide (50 μg/ml). Stained slides were evaluated using a fluorescence microscope and the Comet IV software (Perceptive Imaging). Data were expressed as Olive Tail Moment (OTM), which represents the percentage of DNA in the tail multiplied by the length between the center of the head and tail. The experiments were repeated three times.

### 4.4 Statistical analysis

Results are recorded as mean ± SD for at least three independent experiments. The Student's *t* test was used for continuous variables. The χ^2^ test was used to examine the relationship between miR‐125a‐5p injection and embryo development. For statistical correlation, Pearson's correlation coefficient was used according to requirements. Statistical analyses were performed using the SPSS software package (version 16.0; IBM SPSS). A *p*‐value < 0.05 was considered statistically significant.

## CONFLICT OF INTEREST

The authors declare that they have no conflict of interest.

## AUTHOR CONTRIBUTIONS

B.Y. and J.M. contributed to the conception and design of the study. K.L., L.Y. L.Z., and S.W. performed the experiments, analyzed the data, and drafted the manuscript. L.Z., Z.Q., CW.L., J.J., Y.Y., W.Y., T.X., Q.C, and S.C. participated in performing the experiments and were involved in data acquisition and analysis. Y.G. and X.C. provided and analyzed the clinical samples. R.M. and L.C. reviewed the manuscript.

## Supporting information

Figure S1

Figure S2

Figure S3

Figure S4

Figure S5

Table S1

Table S2

Supplementary Material

## Data Availability

The data that support the findings of this study are available from the corresponding author upon reasonable request.
