# Supplementary figures and images for: miR‐125a‐5p increases cellular DNA damage of aging males and perturbs stage‐specific embryo development via Rbm38‐p53 signaling
Source: Aging Cell. 2021 Nov 9;20(12):e13508. doi: 10.1111/acel.13508 (PMC8672779; doi:10.1111/acel.13508)

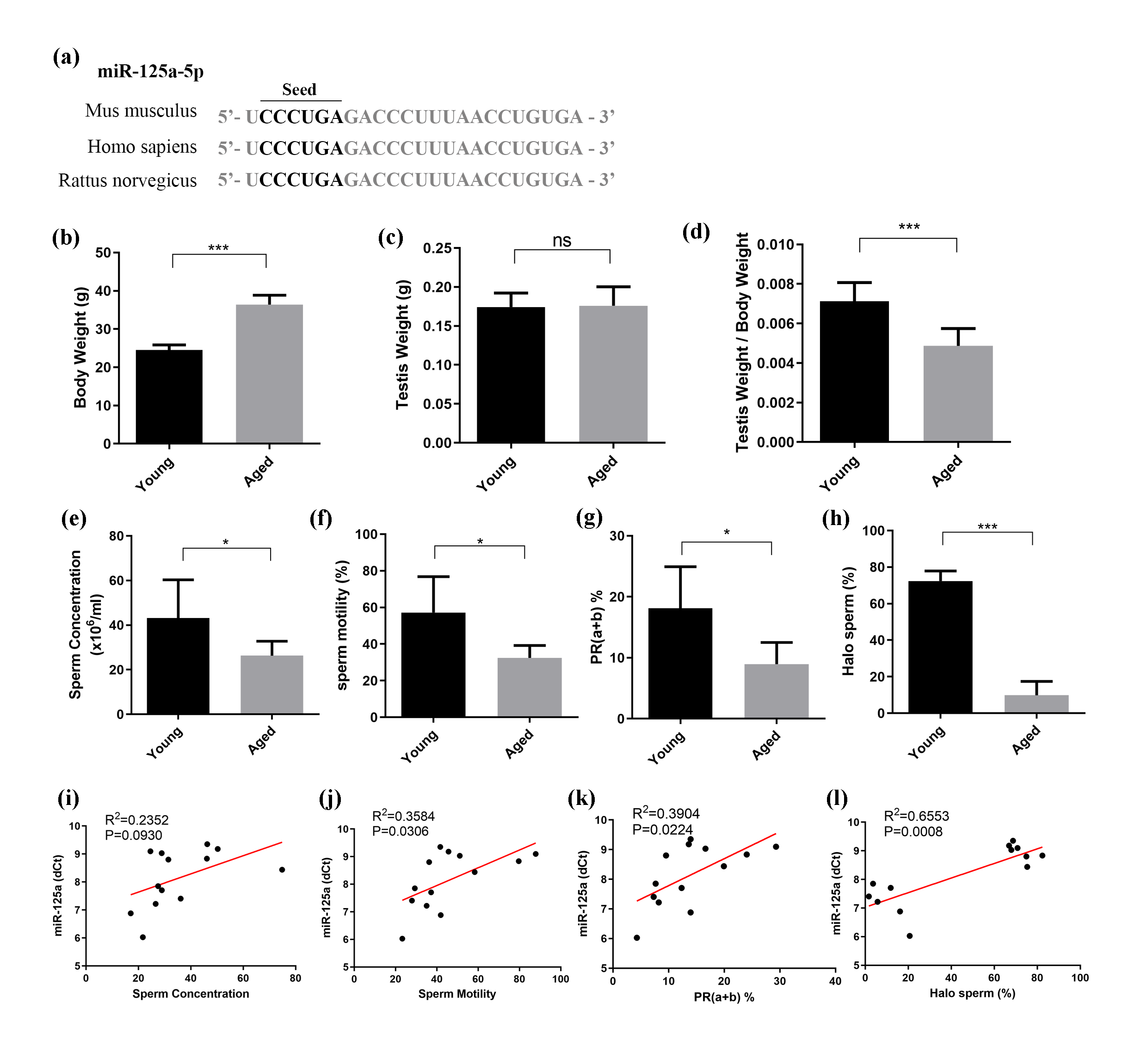

Supplement: Supplementary file 1 — Figure S1 [file ACEL-20-e13508-s002.tif]

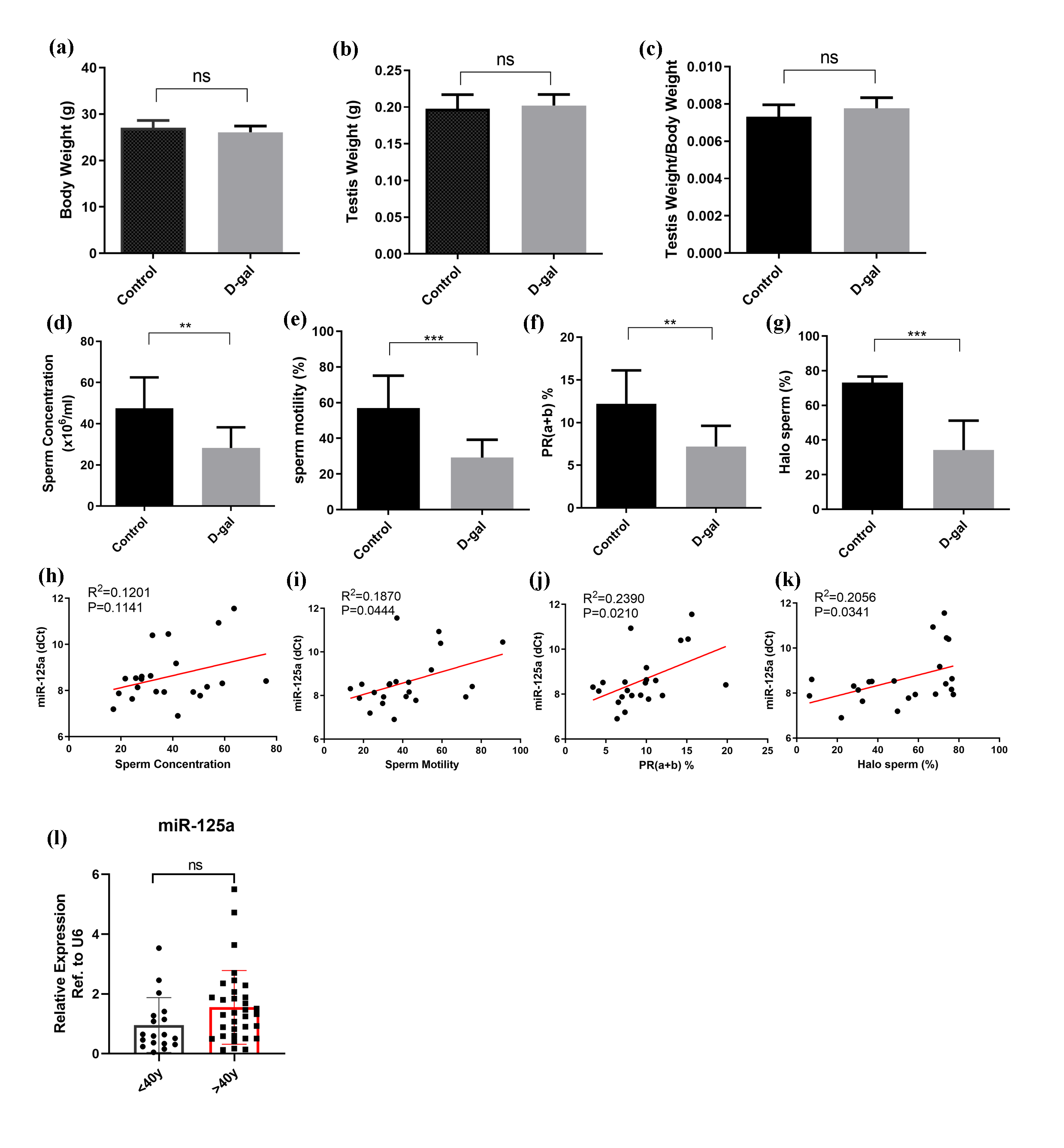

Supplement: Supplementary file 2 — Figure S2 [file ACEL-20-e13508-s003.tif]

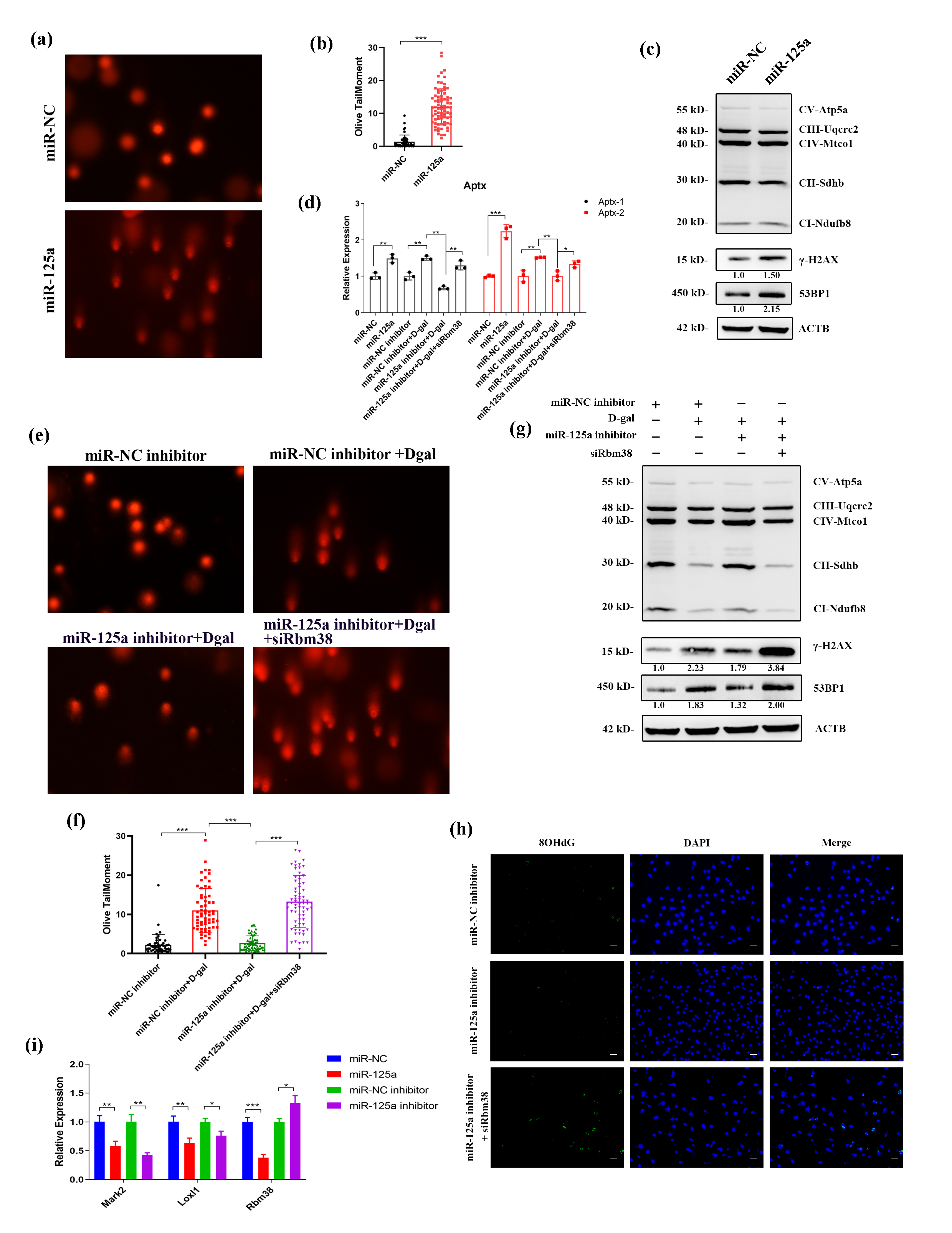

Supplement: Supplementary file 3 — Figure S3 [file ACEL-20-e13508-s006.tif]

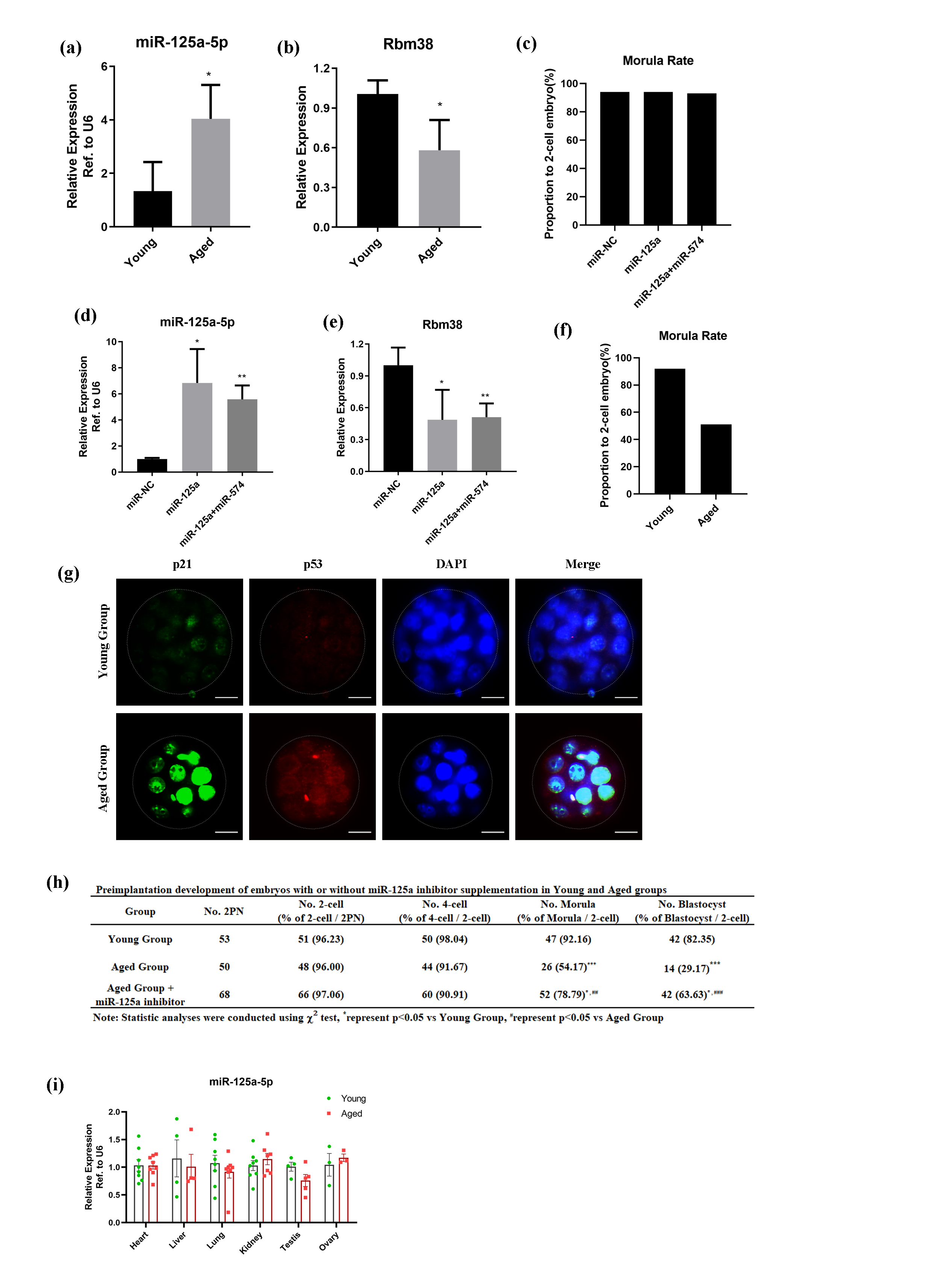

Supplement: Supplementary file 4 — Figure S4 [file ACEL-20-e13508-s004.tif]

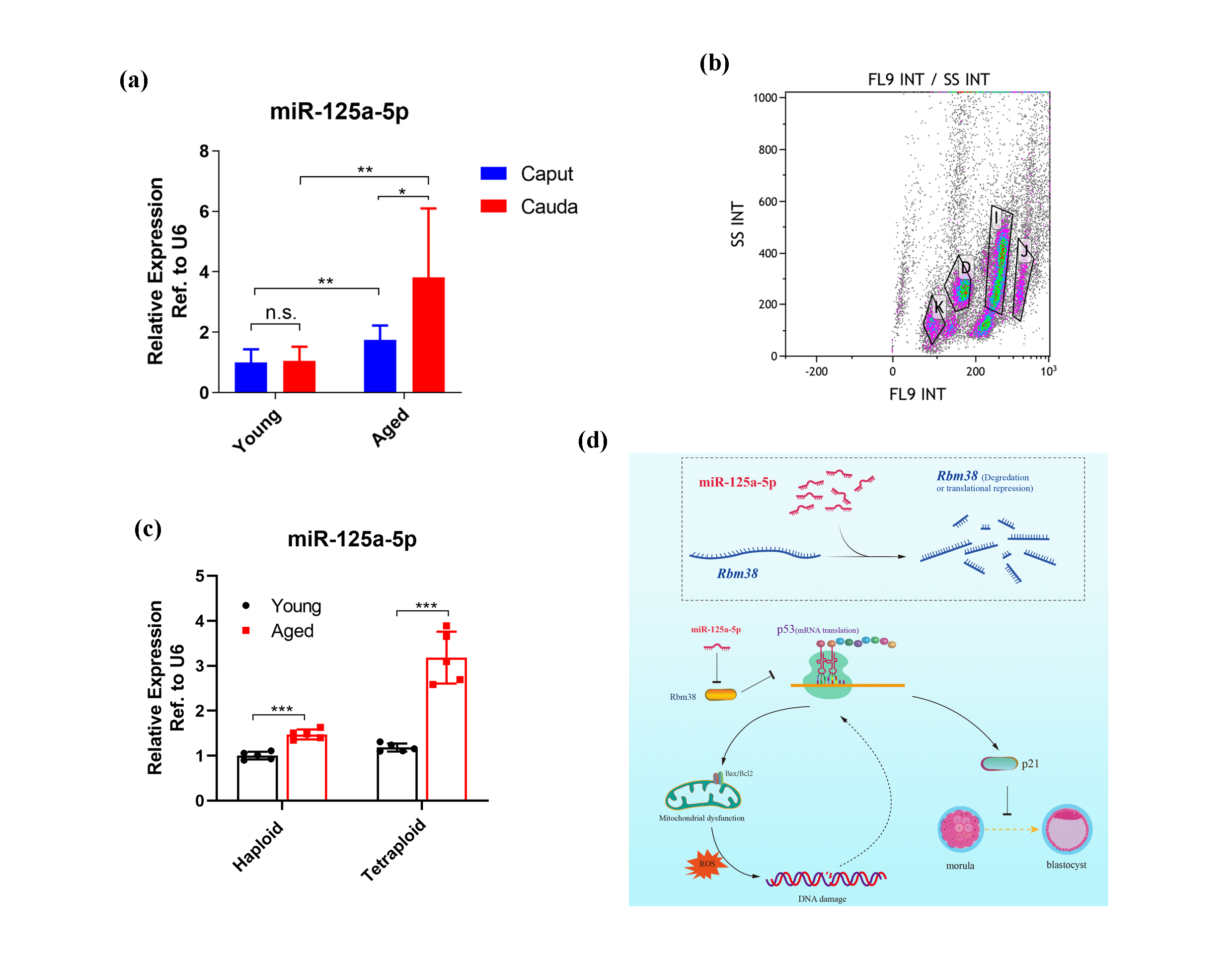

Supplement: Supplementary file 5 — Figure S5 [file ACEL-20-e13508-s005.tif]
