# Supplementary material for: miR‐125a‐5p increases cellular DNA damage of aging males and perturbs stage‐specific embryo development via Rbm38‐p53 signaling
Source: Aging Cell. 2021 Nov 9;20(12):e13508. doi: 10.1111/acel.13508 (PMC8672779; doi:10.1111/acel.13508)
Supplement: Supplementary file 6 — Table S1 [file ACEL-20-e13508-s001.docx]

**Oligonucleotide Sequences used in this study**

| **Gene Description** | **Forward** | **Reverse** |
| --- | --- | --- |
| **β-actin-q** | 5'-AGCCATGTACGTAGCCATCC-3' | 5'-CTCTCAGCTGTGGTGGTGAA-3' |
| **Rbm38-q** | 5'-GACGCATCGCTCAGAAAGT-3' | 5'-GAGGAGTCAGCCCGTAGGT-3' |
| **Loxl1-q** | 5'-GCCCATCTGTACTCCTTGC-3' | 5'-TCGTAGTGGCTGAACTCGTC-3' |
| **Mark2-q** | 5'-GGCGGACAGAGTTGATGG-3' | 5'-TGAACCTTGTGGGATGGAG-3' |
| **Aptx-1-q** | 5'-TGCAGGTTTACAAAGACG-3' | 5'-AAGGCAAGGAGAATCAAA-3' |
| **Aptx-2-q** | 5'-AATGCAGGTTTACAAAGACG-3' | 5'-CAGACTGGAAATGGAGGC-3' |
| **Rbm38-siRNA1** | 5'-GCAUCGCUCAGAAAGUACUTT-3' | 5'-AGUACUUUCUGAGCGAUGCTT-3' |
| **Rbm38-siRNA2** | 5'-CCCUAACCCUAUCAUCGAUTT-3' | 5'-AUCGAUGAUAGGGUUAGGGTT-3' |
| **Rbm38-RIP-1** | 5'-ACGGGCTTTGCTGTTGGT-3' | 5'-CGAGGACAGTGACGGGACA-3' |
| **Rbm38-RIP-2** | 5'-ACCACCATTTGGGTCTCCT-3' | 5'-CCAGAGTGAGTGTCCCTTCC-3' |
| **Rbm38-RIP-3** | 5'-GGCTGCCATTACCAGACCAC-3' | 5'-CAGGTGACAACGGGAGGGT-3' |
| **Rbm38-WT** | 5'-GCGCTCGAGCAGTTGCCCCTCAGT  CGAA-3' | 5'-AATGCGGCCGCTAACAGGCATGCTGT  GGAGTTC-3' |
| **Rbm38-Mut** | 5'- GCGCTCGAGCAGTTGCCCCTCAGT  CGAA -3' | 5'- AATGCGGCCGCTAACAGGCATGCTGT  GGAGTTCCCGCCCGCACACTGGCTGGG  ACTCGTAGGTGTGGAGGGAC -3' |
