# Supplementary material for: miR‐125a‐5p increases cellular DNA damage of aging males and perturbs stage‐specific embryo development via Rbm38‐p53 signaling
Source: Aging Cell. 2021 Nov 9;20(12):e13508. doi: 10.1111/acel.13508 (PMC8672779; doi:10.1111/acel.13508)
Supplement: Supplementary file 8 — Supplementary Material [file ACEL-20-e13508-s008.docx]

**Supplementary experimental procedures**

**Clinical sample collection**

All clinical samples were collected according to protocols approved by the Medical Ethics Committee of Nanjing University, Jinling Hospital. All patients signed informed consent for the collection and use of their samples in this study. Those with a history of cryptorchidism, vascular trauma, orchitis, obstruction of the vas deferens, vasectomy, abnormalities in chromosome number or microdeletions of the azoospermia factor region on the Y chromosome were excluded from the study. Semen specimens were collected by masturbation after a period of 2-7 days of sexual abstinence and were kept to liquefy at 37 °C for 30 min. After liquefaction, semen parameters were analyzed using a computer-aided sperm analysis (CASA) system (CFT-9201; Jiangsu Rich Life Science Instrument Co., Ltd., Nanjing, China). Sperm were obtained by centrifuging semen samples at room temperature at 2000 rpm for 5 min, then washed with PBS for 3 times and stored at -80 °C until use.

**Computer-assisted sperm analysis**

Epididymal sperm were prepared by computer-assisted semen analysis as described previously. Briefly, the epididymis was dissected, and the sperm inside were squeezed out with forceps. After incubation in human tubal fluid medium (500 μl per epididymis) at 37 °C for 30 min, sperm were subjected to motility analyses using a Sperm Quality Analyzer. For each measurement, a suspension of spermatozoa was loaded into a microchamber slide with 100 μm depth. 300 spermatozoa were analyzed using the standard setting.

**Sperm DNA fragmentation analysis**

The fragmentation of sperm DNA was measured by Sperm Chromatin Dispersion test (SCD). Briefly, the test was performed using the SpermFunc®DNAf-Sperm DNA Fragmentation test kit (BRED Life Science Technology Inc., Shengzhen, China) according to the manufacturer’s protocol. The SCD test is based on the principle that sperm with fragmented DNA fails to produce the characteristic halo of dispersed DNA loops observed in sperm with non-fragmented DNA, following acid denaturation and the removal of nuclear proteins (Fernandez et al., 2003). The percentage of sperm with different halos (small halo, without a halo and degraded are considered to be sperm with fragmented DNA) was calculated and the extent of DNA damage for each semen sample was expressed as the sperm DNA fragmentation index (DFI). 300-500 sperms were counted for each semen sample to calculate the DFI.

**Transient transfection**

The miR-125a-5p mimics and inhibitor were purchased from RiboBio Company (Guangzhou, China). Small interfering RNAs (siRNAs) directed against Rbm38 were designed and synthesized by GenePharma Company (Shanghai, China). Transfection was performed with siRNA or miR-125a-5p mimics/inhibitor and their respective negative control RNAs, using a Lipofectamine 2000 kit (Invitrogen, CA) according to the manufacturer’s instructions. Cells were harvested 24 h after transfection and used as needed.

**ATP measurements**

ATP was measured by an ATP Assay kit (Beyotime, China). Briefly, GC2 cells were collected, washed and lysed after transfection with miR-125a-5p mimics or inhibitor for 24 h. ATP was detected according to the manufacturer’s instructions. The protein level of the lysate was measured with an Enhanced BCA Protein Assay Kit (Beyotime, China). The relative ATP level was calculated according to the following formula: relative ATP level=ATP value/protein value. All experiments were performed in three replicates.

**Measurements of Reactive Oxygen Species (ROS)**

The ROS was measured by an ROS Detection Kit (Jiancheng Bioengineering Institute, Nanjing, China). Briefly, GC2 cells were transfected with miR-125a-5p mimics or inhibitor for 24 h and then loaded with 1 μM 2,7-dichlorofuorescin diacetate (DCFH-DA) for 0.5 h. The procedure was conducted at 37 °C. The cells were subsequently rinsed three times with PBS, and the images were captured with a fluorescence microscope (IX73; Olympus Corporation, Tokyo, Japan).

**Flow cytometry detection of Mitochondrial Membrane Potential**

Flow cytometry was used to analyze mitochondrial membrane potential (MMP). Briefly, GC2 cells were harvested after transfection of miR-125a mimics or inhibitor for 24 h, and then stained with JC-1 according to the manufacturer’s instructions. The mitochondrial membrane potential detection kit (Beyotime, China) was used for cell staining. The stained cells were detected by a FACS Caliber (Becton Dickinson, Mountain View, NJ, USA) and analyzed using Modfit LT software (Becton Dickinson, Mountain View, NJ, USA). All experiments were performed in three replicates.

**Immunofluorescence**

GC2 cells were transfected with miR-125a-5p mimics or inhibitor for 24 h, fixed with 4% paraformaldehyde for 10 min, permeabilized with 0.1% Triton X-100 for 20 min, blocked in 1% BSA-supplemented PBS, and then incubated overnight at 4 °C with anti-8-OHdG antibody (Abcam, Cambridge, MA, USA). The following day, the samples were washed with PBS for 5 min three times and incubated with anti-rabbit IgG H&L secondary antibody for 1 h in the dark. DAPI was added to each dish and incubated for 10 min in the dark at room temperature. The intensity of 8-OHdG was visualized by a fluorescence microscope (IX73; Olympus Corporation, Tokyo, Japan).

Embryos were fixed with 4% paraformaldehyde for 30 minutes and then permeabilized with 0.5% Triton X-100 for 20 minutes. After 1 h of blocking in 1% BSA-supplemented PBS, samples were incubated overnight at 4 °C with Rabbit monoclonal anti-p21 (1:50, cat. no., ab188224) and mouse monoclonal anti-p53 (1:50, cat. no., ab26) antibody. After three washes in PBST, embryos were incubated with FITC-conjugated goat anti-rabbit IgG (H+L), and TRITC-conjugated goat anti-mouse IgG (H+L) (ZSGB-BIO) for 1 h at room temperature. After washing in PBST three times, embryos were stained with DAPI for 20 min. Then embryos were transferred to glass slides and observed using a laser scanning confocal microscope (LSM 800, CarlZeiss).

**Western blotting**

Western blotting was performed as described previously (Wang et al., 2020). Briefly, proteins were harvested as indicated in the figure legends, separated by sodium dodecyl sulfate polyacrylamide gel electrophoresis (SDS-PAGE) and transferred onto PVDF membranes. The primary antibodies include: Rabbit polyclonal anti-bax (cat. no., ab69643), Rabbit polyclonal anti-bcl2 (cat. no., ab194583), Rabbit monoclonal anti-p53 (cat. no., ab26), Rabbit polyclonal anti-p53 (phosphor S15) (cat. no., ab1431), Rabbit polyclonal anti-53PB1 (cat. no., ab175933), Rabbit polyclonal anti-53PB1 (cat. no., ab175933), Total OXPHOS Rodent WB antibody Cocktail (ab110413) and Rabbit polyclonal anti-gamma H2A.X(ab81299) from Abcam (Cambridge, MA, USA); mouse monoclonal anti-Rbm38 (cat. no., SC-365898) from Santa Cruz; Rabbit polyclonal anti-p21 (cat. no., 27296-1-AP) from Proteintech, and mouse monoclonal anti-β-actin (cat. no., 3700S) from Cell Signaling Technology. After primary antibody incubation, the blots were then incubated with HRP-conjugated secondary antibodies for 1 h at room temperature, prior to being processed using an ECL Plus Western Blotting Detection System. β-actin was used as a loading control for Western blotting.

**Luciferase reporter assay**

For miRNA target validation, the targeted sequence of Rbm38 was PCR-amplified using mouse cDNA and cloned into the *XhoI-NotI* site downstream of the pmiR-RB-Report^TM^ vector (RiboBio, Guangzhou, China). A construct containing mutated miRNA binding sites (seed sequence) in Rbm38 was PCR-amplified and cloned into the pmiR-RB-Report^TM^ vector (RiboBio, Guangzhou, China). GC2 cells were cultured in 24-well plates in DMEM supplemented with 10% FBS at 37 °C, and then co-transfected with wild type or mutated construct (200 ng/well) and miR-125a-5p mimics (20 nM) using Lipofectamine 2000 reagent. At 24 h post-transfection, cell lysates were analyzed for luciferase activity using the Duo-Lite Luciferase Assay System (Vazyme Biotech Co., Ltd, Nanjing, China) according to the manufacturer’s protocol. Data were normalized against the Renilla luciferase values.

**RNA immunoprecipitation**

GC2 cells were transfected with miR-125a-5p mimics for 48 h, and RNA immunoprecipitation (RIP) experiments were performed using the Magna RIP™ RNA-Binding Protein Immunoprecipitation Kit (Millipore, USA) according to the manufacturer’s instructions. To demonstrate that the detected signals were from the RNA that was specifically bound, total RNA (input controls) and corresponding species IgG controls were performed simultaneously. Immunoprecipitations of Ago2 was performed using an anti-Ago2 antibody (Abcam) overnight at 4 °C. RNAs were extracted by phenol:chloroform:isoamyl alcohol method and subjected to qRT-PCR.

**Embryos collection, culture and miRNA microinjection**

6-8-week-old female ICR mice received an injection of human chorionic gonadotrophin (hCG) 2 d after 5 IU Pregnant Mares Serum Gonadotropin (PMSG) priming and were then mated overnight with 6-8-weeks-old (Young Group) or 13-month-old (Aged Group) male ICR mice. Zygotes were obtained by flushing the oviducts 20 h after the hCG injection. Embryos were cultured in KSOM (Millipore, Massachusetts, USA) medium under mineral oil at 37 °C in a 5% CO_2_, 5% O_2_ and 90% N_2_ incubator.

Microinjection of miR-125a-5p, miR-125a/miR-574 mimics, or miR-125a-5p inhibitor was performed with a Narishige microinjector. The miRNA mimics and inhibitor were diluted with water to give a stock concentration of 100 nM and 2 μM, and then a 2.5 picoliter solution was injected into embryos. MiR-NC was injected as a control.

**Isolation of spermatogenic cells**

Germ cell isolation was performed as described previously (Lima et al., 2017). The testes from mice were collected in PBS and placed on ice. After removal of the tunica albuginea, the testes were incubated in 5 mL of PBS containing 120 U/mL of collagenase type I at 32 ^o^C with gentle agitation for 5 min. The dispersed seminiferous tubules were further digested with 5 mL of 0.25% trypsin, 0.1 mL of DNase I (5 mg/mL) by pipetting gently several times at 32 ^o^C for 8 min, and then terminated by adding 0.5 mL of fetal bovine serum (FBS) to inactivate trypsin. Following two-step enzymatic digestion, the dissociated testicular cell suspension was then filtered through a PBS-prewetted cellular filter with pore size of 70 μm. The cell suspension was centrifuged at 500 g for 5 min at 4 ^o^C, and the supernatant was carefully removed from the pellet. The cells in the pellet were resuspended at a concentration of 1 x 10^6^ cells/mL in DMEM with Hoechst 33342 (3 mg/mL) and 5 μl DNase I. For FACS, the cell suspension was rotated for 20 min at 32 ^o^C in the oven at 10 r.p.m./min speed, then centrifuged at 500 g for 5 min at 4 ^o^C, and resuspended in 0.3ml DMEM for sorting. Cell populations were collected based on their fluorescent label with Hoechst 33342 staining using FACS.

**References**

Fernandez, J. L., Muriel, L., Rivero, M. T., Goyanes, V., Vazquez, R., & Alvarez, J. G. (2003). The sperm chromatin dispersion test: a simple method for the determination of sperm DNA fragmentation. *Journal of andrology, 24*(1), 59-66.

Lima, A. C., Jung, M., Rusch, J., Usmani, A., Lopes, A. M., & Conrad, D. F. (2017). A Standardized Approach for Multispecies Purification of Mammalian Male Germ Cells by Mechanical Tissue Dissociation and Flow Cytometry. *J Vis Exp*(125). doi:10.3791/55913

Wang, S., Chen, Q., Zhang, Y., Zheng, F., Xue, T., Ge, X., . . . Yao, B. (2020). Omega-3 polyunsaturated fatty acids alleviate hydrogen sulfide-induced blood-testis barrier disruption in the testes of adult mice. *Reprod Toxicol, 98*, 233-241. doi:10.1016/j.reprotox.2020.10.007
